# Apple Flavonoids Suppress Carcinogen-Induced DNA Damage in Normal Human Bronchial Epithelial Cells

**DOI:** 10.1155/2017/1767198

**Published:** 2017-06-18

**Authors:** Vazhappilly Cijo George, H. P. Vasantha Rupasinghe

**Affiliations:** ^1^Department of Plant, Food, and Environmental Sciences, Faculty of Agriculture, Dalhousie University, Truro, NS, Canada; ^2^Department of Pathology, Faculty of Medicine, Dalhousie University, Halifax, NS, Canada

## Abstract

**Scope:**

Human neoplastic transformation due to DNA damage poses an increasing global healthcare concern. Maintaining genomic integrity is crucial for avoiding tumor initiation and progression. The present study aimed to investigate the efficacy of an apple flavonoid fraction (AF4) against various carcinogen-induced toxicity in normal human bronchial epithelial cells and its mechanism of DNA damage response and repair processes.

**Methods and Results:**

AF4-pretreated cells were exposed to nicotine-derived nitrosamine ketones (NNK), NNK acetate (NNK-Ae), methotrexate (MTX), and cisplatin to validate cytotoxicity, total reactive oxygen species, intracellular antioxidants, DNA fragmentation, and DNA tail damage. Furthermore, phosphorylated histone (*γ*-H2AX) and proteins involved in DNA damage (ATM/ATR, Chk1, Chk2, and p53) and repair (DNA-PKcs and Ku80) mechanisms were evaluated by immunofluorescence and western blotting, respectively. The results revealed that AF4-pretreated cells showed lower cytotoxicity, total ROS generation, and DNA fragmentation along with consequent inhibition of DNA tail moment. An increased level of *γ*-H2AX and DNA damage proteins was observed in carcinogen-treated cells and that was significantly (*p* ≤ 0.05) inhibited in AF4-pretreated cells, in an ATR-dependent manner. AF4 pretreatment also facilitated the phosphorylation of DNA-PKcs and thus initiation of repair mechanisms.

**Conclusion:**

Apple flavonoids can protect in vitro oxidative DNA damage and facilitate repair mechanisms.

## 1. Introduction

Mammalian genomic DNA is susceptible to various environmental, cytotoxic, or genotoxic agents that sense DNA damage and activate signaling cascades for effective repair mechanisms. Under a normal circumstance with a specific type of DNA lesion, DNA damage is commonly repaired through nonhomologous end joining (NHEJ)/homologous recombination (HR) mechanisms [1, 2]. Alkylating agents, platinum drugs, antimetabolites, topoisomerase inhibitors and ionizing radiations, nitrosoureas, aziridine compounds, alkyl sulphonates, and triazine compounds are some of the electrophiles that covalently transfer alkyl-groups onto the DNA bases, disrupting the DNA helix and induces DNA breaks [3]. DNA double-strand breaks (DSBs) are the most lethal lesions that can result in mutations, chromosomal aberrations, and cell death [4, 5]. Extensive DNA damage and defects in repair systems can lead to poor genomic stability and initiate cardiovascular disease and cancer [2, 6]. Hence, maintaining genomic integrity possess global healthcare challenge and should be well addressed.

An increased level of oxidative stress often causes excessive reactive oxygen species (ROS) generation, which breaks the equilibrium of metabolic process of normal cells and initiates DSBs [7]. As a result, the cells activate DNA damage response (DDR) mechanisms and initiate various enzymes that modify the DNA and nuclear damage. Recruitment of phosphatidylinositol-3-kinase (PI3K) family members to the site of DNA damage is the first step of DDR mechanisms, and the phosphorylation of ataxia telangiectasia-mutated (ATM) or ATM-Rad3-related (ATR) kinases are often followed in DDR process [8]. The phosphorylation of ATM/ATR regulates downstream targets including cell cycle check point kinases (Chk2/Chk1), tumor suppressor p53, and phosphorylated histone *γ*-H2AX foci, commonly known as a marker for DSBs [9]. *γ*-H2AX foci serve as a platform for the assembly and recruitment of other DNA repair factors, including mediators of DNA damage check point 1 (MDC1) to initiate DDR mechanisms [10]. DNA-dependent protein kinases (DNA-PK), composed of Ku70/80 heterodimer and a catalytic subunit (DNA-PKcs), serve as the pinnacle protein that cooperates with ATR/ATM to phosphorylate other proteins involved in the DNA damage [11, 12]. Upon phosphorylation in serine and threonine residues (T2609, T3950, and S2056), DNA-PK initiates NHEJ repair mechanisms which are found to be very common in mammalian cells [4]. DNA-PK also gets autophosphorylated and expressed differentially in normal and malignant human tissues with relatively little variation in level [13]. However, there are many other proteins involved in this complex mechanisms and their roles are still inconclusive.

Development of effective nutraceuticals from natural resources has been major research endeavors over the past decade. While several reports are available to show the protective effects of various plant flavonoids and extracts against different genotoxicity [14], to the best of our knowledge, there are no specific studies available to show the mechanism of action of apple flavonoids to exert protection against DNA damage in normal human cells. Our previous studies have shown that an apple peel flavonoid fraction (AF4) possess antioxidant, neuroprotective, anti-inflammatory, and anticancer activities in various in vitro and in vivo models [15–17]. Moreover, AF4 is highly rich with flavonoids and phenolic acids such as quercetin glycosides, cyanidin 3-galactoside, epicatechin, phloridzin, and chlorogenic acid [17]. In light of these findings, we hypothesized that AF4 could possibly render protection against DNA damage induced by various chemicals or environmental agents, whose primary target is inevitably airway epithelial cells in the lung. To test this hypothesis, we investigated the effects of AF4 on normal human bronchial epithelial cells (BEAS-2B) challenged with known carcinogenic chemical agents such as 4-(methylnitrosamino)-1-(3-pyridyl-d4)-1-butanone (NNK), 4-[(acetoxymethyl) nitrosamino]-1-(3-pyridyl)-1-butanone (NNK acetate; NNK-Ae), methotrexate (MTX), and cisplatin. We also analyzed the signaling proteins involved in DNA damage pathways since understanding the DNA repair mechanisms has important implication in developing a potent therapeutic agent.

## 2. Material and Methods

### 2.1. Chemicals, Kits, and Antibodies

Bronchial Epithelial Cell Growth Medium (BEGM) for BEAS-2B cells was purchased from Lonza (Walkersville, MD, USA). COMET SCGE assay kit was purchased from ENZO (New York, NY, USA). Cellular DNA fragmentation ELISA kit was purchased from Roche Diagnostics (Berlin, Germany). For immunofluorescence studies, anti-H2AX primary antibody (S139) was obtained from Millipore (Etobicoke, ON, Canada) and secondary antibody Alexa Flour 594 donkey anti-mouse from Life Tech (Carlsbad, CA, USA). Bicinchoninic acid (BCA) protein assay kit was purchased from Thermo Scientific (Chelmsford, MA, USA). The total antioxidant capacity (TAC) kit was purchased from Biovision (Milpitas, CA, USA). Antibodies for DNA-PK, p-ATM, p-ATR, p-Chk1, p-Chk2, p-H2AX, p-P53, Ku80, SOD1, catalase, GPX1, and beta-actin were purchased from Cell Signaling Technology (Danvers, MA, USA). p-DNA-PKcs antibody was purchased from Abcam (Toronto, ON, Canada). DNA-PK inhibitor [NU7026; (2-(morpholin-4-yl)-benzo[h]chomen-4-one)] was purchased from Sigma-Aldrich (Oakville, ON, Canada). NNK and NNK-Ae were purchased from Toronto Research Chemicals (Toronto, ON, Canada). Cisplatin, MTX, and NP-40 were purchased from Sigma-Aldrich (Oakville, ON, Canada). Apple flavonoid fraction (AF4) was isolated from apple peels as described previously [14]. Stock solutions were prepared in 100% dimethyl sulfoxide (DMSO), and the final concentrations never exceeded 0.5% (*v*/*v*) in culture treatment medium.

### 2.2. Cell Culture

Normal human bronchial epithelial cells (BEAS-2B) were purchased from American Tissue Type Culture Collection (ATCC; CRL-9609) and were cultured in BEGM media at 37°C in a humidified incubator with 5% CO_2_. Cells were cultured on polystyrene T75 (75 cm^2^) culture flasks, precoated with a mixture of 0.01 mg/mL fibronectin, 0.03 mg/mL bovine collagen type I, and 0.01 mg/mL bovine serum albumin dissolved in BEBM (basal) medium for overnight. Cells were grown to ~70% confluence during all experimental conditions and were used from early passages (<10) and within exponential growth phase.

### 2.3. Cell Viability by MTS Assay

Cell Titer 96™ aqueous cell viability assay (MTS) [18] was used to perform the viability of BEAS-2B cells under different treatment conditions. In order to find out the sublethal dose for AF4, a dose-dependent preliminary assay for various concentrations of AF4 was performed for 24 h. Similarly, the dose-response effect for various carcinogens (NNK, NNK-Ae, cisplatin, and MTX) was also standardized using this assay. For cytoprotection analysis, 1 × 10^4^ cells were plated on a 96-well plate with media of 150 *μ*L/well. After 24 h, cells were either pretreated with AF4 (50 *μ*g/mL) prior to different carcinogen treatments (200 *μ*M NNK; 100 *μ*M NNK-A; 10 *μ*M cisplatin; and 200 *μ*M MTX) or alone with carcinogens for additional 24 h. Fifteen microliters of MTS reagent (with PMS) was then added to each well and incubated further 3 h at dark. Absorbance was recorded at 490 nm using a microplate reader (Infinite® 200 PRO, TECAN, Switzerland). DMSO control cells which are devoid of any treatments and cells containing only culture medium and MTS reagent served as the blank for each experiment.

### 2.4. Measurement of Intracellular ROS

The ROS level was measured in BEAS-2B cells after treatments as described previously [19]. 2′,7′-Dichlorofluorescin diacetate (DCFH-DA) is readily taken up by cells and is subsequently hydrolyzed to DCFH, which can be oxidized to measurable fluorescent product dichlorofluorescein (DCF). AF4-pretreated cells (for 1 h) were exposed to 3 h of carcinogens or alone in different experimental groups. Cells with only DMSO media served as the vehicle control. After treatments, DCFH-DA was added to the cell culture plates at a final concentration of 5 *μ*M followed by 40 min incubation at dark. The fluorescence degradation was then measured at an excitation wavelength of 490 nm and an emission wavelength of 510 nm by using Infinite 200 PRO, TECAN, Switzerland. The results were expressed as relative total ROS level with respect to DMSO control.

### 2.5. Total Antioxidant Capacity (TAC)

A colorimetric-based method was used to measure intracellular TAC, according to the manufacturer's instructions with slight modification. Briefly, the total cell lysate was prepared after treatments in NP-40 lysis buffer (5 M NaCl, 1 M Tris, 10% NP-40). Each sample was added with 100 *μ*L of freshly prepared Cu^2+^ working solution and incubated for 1.5 h at dark. The reduction (Cu^2+^ to Cu^+^) reaction was then measured at 570 nm by using Infinite 200 PRO, TECAN, Switzerland. Trolox was used as the standard to quantify the TAC of the tested samples, and the results were expressed in Trolox equivalence.

### 2.6. *γ*-H2AX Immunofluorescence Assay

Immunofluorescence method [20] was used to measure the DNA damage at histone level by quantifying *γ*-H2AX foci in BEAS-2B cells. Briefly, 2 × 10^5^ cells were seeded on a coated cover slip placed in a 6-well plate with 24 h incubation. For experimental set-up, the cells were then treated with AF4 alone for 1 h or prior to each carcinogen treatment for 3 h (same treatment conditions were maintained for all following experiments). DMSO media served as a control for each test sample. After treatments, cells were washed thoroughly with PBS and fixed in 3.7% formaldehyde for 20 min at dark. The cells were then permeabilized with 0.5% Triton X-100 in PBS for 15 min on a rocker at room temperature followed by blocking with 4% BSA for 20 min. The cells were incubated with primary antibody (1 : 250) for 1 h at room temperature, washed three times with PBS, and then incubated with secondary antibody (1 : 500) for 45 min. After washing the cells three times in PBS, coverslips were carefully transferred into slides and mounted by using wet-mounting medium, Vectashield® containing DAPI and sealed with nail polish. The fluorescent images were then captured by using a microscopy (ZEISS, X-Cite series 120 PC) at 100x magnification.

### 2.7. DNA Fragmentation Analysis

DNA fragmentation in BEAS-2B cells was measured by cellular DNA fragmentation ELISA kit [21] as per the supplier's instructions. In short, BEAS-2B cells were labeled with 10 *μ*M bromodeoxyuridine (BrdU) at 1 × 10^5^ cells/mL density. Hundred microliters of BrdU-labelled cells in culture medium were treated as per above-mentioned conditions. The cells were then lysed with lysis buffer, and apoptotic DNA fragments in supernatants were collected for each sample after centrifugation at 270*g* for 10 min. Hundred microliters of the sample was then transferred to precoated anti-DNA 96-well, flat-bottom microplates with incubation for 90 min at 25°C. The DNA was then denatured by microwave irradiation (500 W for 5 min) followed by the addition of 100 *μ*L anti-BrdU-POD conjugate solution with additional 90 min of incubation. The plates were washed by three times with wash buffer (1x), and 100 *μ*L of substrate (TMB) solution was then added for color development. Twenty-five microliters of stop solution was added after 5 min, and the plates were read at 450 nm using a microplate reader (Infinite 200 PRO, TECAN, Switzerland).

### 2.8. Comet Assay

The comet assay was performed to measure the DNA tail moment as per kit instructions with minor modifications. After treatments, 1 × 10^5^ cells were combined with molten LMA (low melting agarose) at a ratio of 1 : 10 (*v*/*v*) and 75 *μ*L of each sample was pipetted on to a comet slide and incubated in dark at 4°C for 20 min. The slides were then immersed in cold lysis buffer at 4°C for 45 min followed by alkaline treatment (300 mM NaOH, 1 mM EDTA, pH > 13) for additional 45 min in dark. The slides were washed with TBE buffer (1x) for 5 min and subjected to horizontal electrophoresis conditions (1 V/cm for 10 min). The slides were air-dried after dipping in 70% ethanol for 5 min, stained with CYGREEN® dye (1 : 1000), and examined under epifluorescence microscopy (ZEISS, X-Cite series 120 PC; Toronto, ON, Canada) with 40x magnification (excitation/emission 489/515 nm). The comets were scored by commercially available software, OpenComet (http://www.cometbio.org), and a minimum of 50 cells was quantified by measuring percentage DNA tail moment.

### 2.9. Western Blotting

The cells were harvested after the treatments and were lysed using 1 × SDS lysis buffer (1 mM Tris–HCl [pH 6.8], 2% *w*/*v* SDS, 10% glycerol) under reduced conditions on the ice. Total protein concentration in each sample was measured by using BCA protein assay kit. A total of 25 *μ*g of protein samples were loaded on 4–12% SDS-PAGE gel and electro-transferred to a nitrocellulose membrane. The membrane was then blocked with 5% nonfat milk solution, probed with specific primary antibodies (1 : 1000) for overnight incubation, washed and reprobed with respective secondary antibodies (1 : 2000) for 45 min, and then developed by enhanced chemiluminescence (ECL) method using Chemidoc MP (Bio-Rad, Mississauga, ON, Canada). Protein expression of each band was normalized with respective actin level, and relative protein expression was quantified with respect to untreated control bands for each experiment.

### 2.10. Statistical Analysis

All the experiments were performed in triplicates (*n* = 3) and for at least three independent times and analyzed by two-tailed Student's *t*-test by using GraphPad Prism software (GraphPad Software Inc., San Diego, CA, USA). Data were presented as mean ± standard deviation (SD), and *p* values ≤ 0.05 were considered as significant between experimental groups.

## 3. Results

### 3.1. Cell Viability and Cytoprotective Effects of AF4

**Figure 1:**
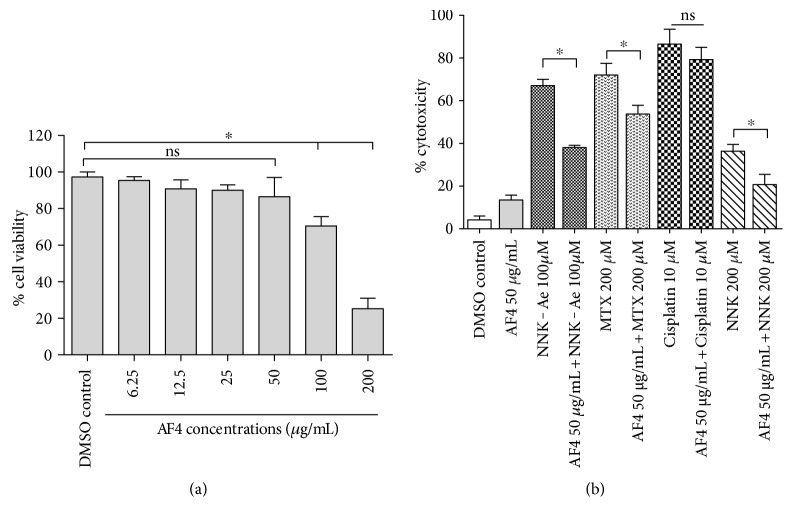
(a) Dose-dependent effect of AF4 on BEAS-2B cells after 24 h of treatment. (b) Cytoprotective effects of AF4 against various carcinogens challenged after 24 h of treatment. Experimental values presented as mean ± SD of *n* = 3 independent experiments. ∗ indicated statistical difference at *P* ≤ 0.05. ns: nonsignificant.

In order to realize the sublethal dosage for AF4, preliminary dose-responsive effects on the viability of BEAS-2B cells were studied using MTS assay. A dose-responsive decline in cell viability was observed in BEAS-2B cells with increasing concentrations of AF4, especially at 100 and 200 *μ*g/mL (Figure 1(a)). However, over ≥80% cell viability was observed up to 50 *μ*g/mL concentrations of AF4 and hence taken for evaluating protective effects in further experiments. Our previous studies have also shown that 50 *μ*g/mL of AF4 did not alter cell viabilities of three primary normal cells treated for 24 and 48 h [17]. DMSO control in all experiments showed ≤5% cytotoxicity. After 24 h of treatments with each carcinogen, we observed a higher cytotoxicity (>50%) for 10 *μ*M of cisplatin, 200 *μ*M of MTX, and 100 *μ*M of NNK-Ae (Figure 1(b)). Cisplatin exhibited a very high cytotoxicity (>80%) among the carcinogens studied. However, NNK did not show higher cytotoxicity for BEAS-2B cells (<50%). Likewise, for studying cytoprotective effects of AF4, we initially treated BEAS-2B cells with AF4 (50 *μ*g/mL) prior to each carcinogen exposure. AF4 pretreatment showed significant (*p* ≤ 0.05) reduction in cytotoxic level for NNK-Ae, MTX, and NNK exposed cells when compared to their treatments alone. In contrast, AF4 pretreatment did not show any significant reduction in cytotoxicity for cisplatin-treated cells and found to be morphologically distinct with rounded-shape or detached cells (data not shown).

### 3.2. ROS Mitigating and Antioxidant Potentials of AF4

**Figure 2:**
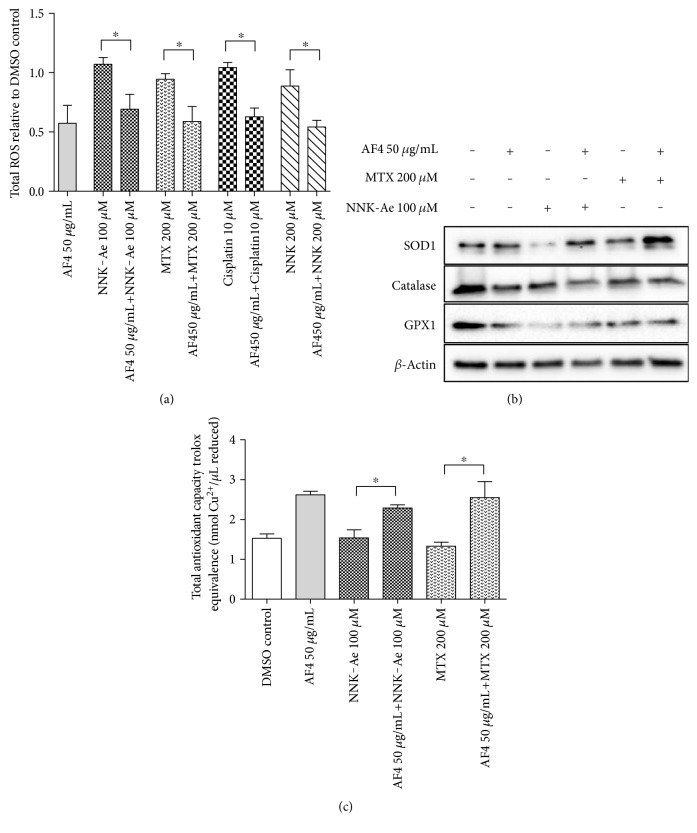
(a) The relative amount of ROS assessed on BEAS-2B cells after exposed to either carcinogen alone or with pretreatment of AF4. (b) Effects of AF4 on intracellular antioxidant enzymes (SOD1, catalase, and GPX1) along with carcinogen-treated groups as shown by western blotting. Beta-actin is used as in internal control to demonstrate equal protein in all tested samples. (c) TAC of BEAS-2B cells after various treatments was measured by a colorimetric kit-based method and showed in Trolox equivalence. Experimental values presented as mean ± SD of *n* = 3 independent experiments. ∗ indicated statistical difference at *P* ≤ 0.05.

Excessive ROS is one of the primary factors that can initiate DNA damage in healthy cells [22]. ROS level was studied either with AF4 alone or with carcinogen-treated BEAS-2B cells, and the data is shown in Figure 2(a). All the carcinogen-treated cells showed an almost two-fold increase in relative to total ROS (DMSO control) levels when compared to AF4-treated cells. Pretreatment with AF4 prior to each carcinogen exposure significantly (*p* ≤ 0.05) reduced ROS levels in these cells. Interestingly, in all the AF4 preexposed cells, we observed similar levels of ROS despite each carcinogen tested in the study.

Antioxidants are well-known for their capacity to mitigate ROS generation, especially under oxidative stress, which is considered as the primary event in many diseases [23]. We assessed the antioxidant enzymes [superoxide dismutase (SOD), glutathione peroxidase (GPX), and catalase] (Figure 2(b)) and TAC (Figure 2(c)) in BEAS-2B cells after treated with either AF4 alone or with carcinogens. Preexposure of AF4 showed an increased SOD1 expression in NNK-Ae or MTX-treated samples when compared to their controls. However, both catalase and GPX levels remained almost the same in all the tested groups. TAC in AF4 preexposed groups showed greater antioxidant capacity than carcinogens alone. The findings indicate that AF4 has enhanced intracellular antioxidant potential.

### 3.3. AF4 Inhibits DNA-Histone Protein Damage

**Figure 3:**
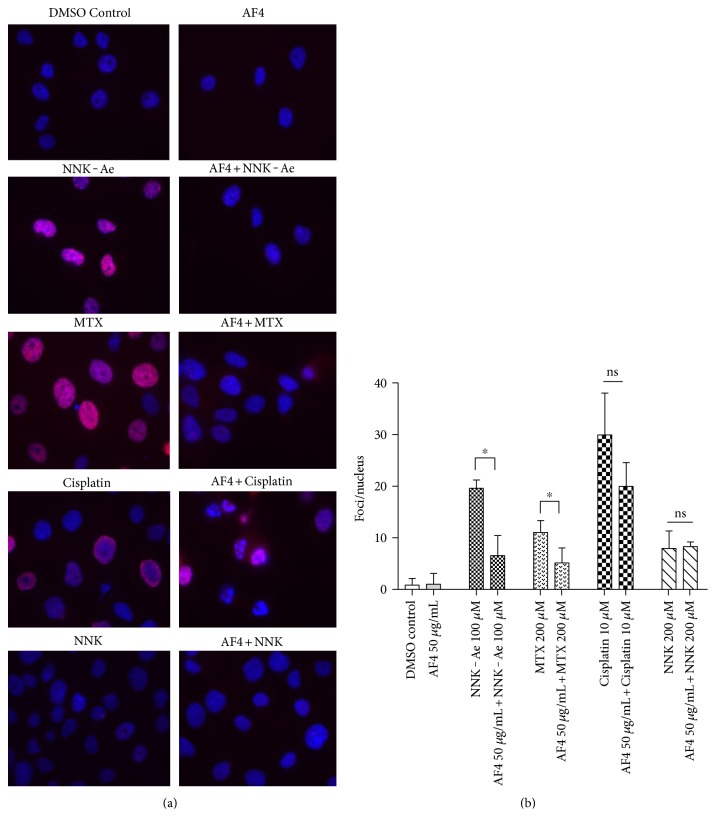
(a) BEAS-2B cells were exposed to either carcinogens alone or in combination with pretreatment of AF4 followed by immunofluorescence staining with *γ*-H2AX antibody and were captured by epifluorescence microscopy at 100x magnification. Nuclei were stained as blue and *γ*-H2AX foci (S 139) appeared as red. The image shown represents cells from three independent experiments. (b) Quantification of focus/nucleus ratio was calculated for each sample from at least 50 cells. ∗ indicated statistical difference at *P* ≤ 0.05.

*γ*-H2AX immunofluorescence assay was used to analyze the DNA damage at histone level after each treatment conditions, and the results are shown in Figure 3(a). DAPI was used to stain the nucleus (blue color) colocalized with *γ*-H2AX foci, which appeared as red color when observed under fluorescence microscope. Cisplatin-, NNK-Ae-, or MTX-treated groups exhibited severe damage at histone level (S 139) when compared to DMSO control cells. Treatment with AF4 did not cause any increase in histone damage level when compared to DMSO control cells. Quantification of data (Figure 3(b)) showed that pretreatment with AF4 significantly (*p* ≤ 0.05) inhibited *γ*-H2AX damage (foci/nucleus) level caused by NNK-Ae or MTX exposure. The DNA damage caused by cisplatin could not able to reduce by preexposure to AF4. As observed in other assays, cisplatin showed the highest damage among all carcinogens tested. Cisplatin and NNK were therefore avoided from all the remaining studies since they are found to be either too toxic or less toxic, respectively, as observed from the *γ*-H2AX assay.

### 3.4. AF4 Protects DNA Fragmentation in BEAS-2B Cells

**Figure 4:**
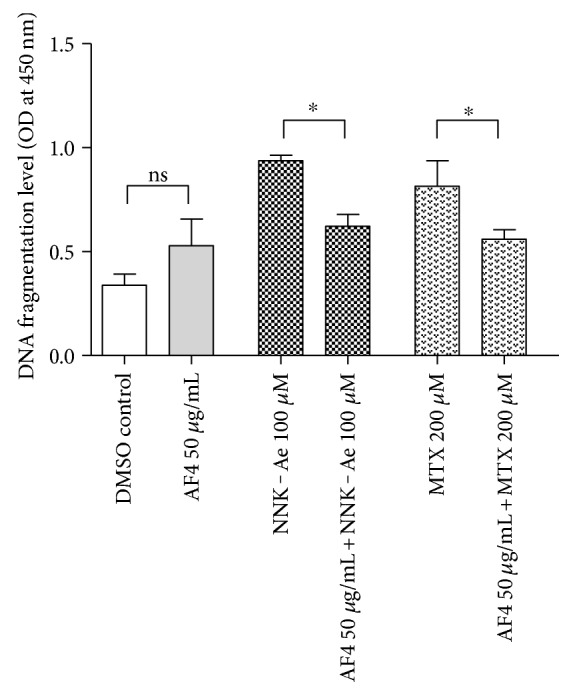
DNA fragmentation level in BEAS-2B cells after exposed to either carcinogen alone or in combination with pretreatment of AF4. OD at 450 nm corresponds to the amount of DNA fragments. Data expressed as mean ± SD of *n* = 3. ∗ indicated statistical difference at *P* ≤ 0.05.

DNA fragmentation was considered as an early event that initiates the phosphorylation of H2AX histone proteins at Serine 139 position [24]. To investigate whether AF4 protects severe toxic effects of NNK-Ae or MTX at DNA level, we used an ELISA method and the fragmentation levels are shown in Figure 4. OD at 450 nm corresponds to the DNA fragmentation levels in BEAS-2B cells. The treatment with NNK-Ae and MTX enhanced the DNA fragmentation levels when compared to DMSO control. We do observe some DNA fragmentation in AF4-treated cells but was found to be nonsignificant with respect to DMSO control. Pretreatment with AF4 significantly (*p* ≤ 0.05) reduced DNA fragmentation in both NNK-Ae- and MTX-treated groups and protect DNA integrity in these cells.

### 3.5. Preexposure to AF4 Reduces DNA Tail Damage

**Figure 5:**
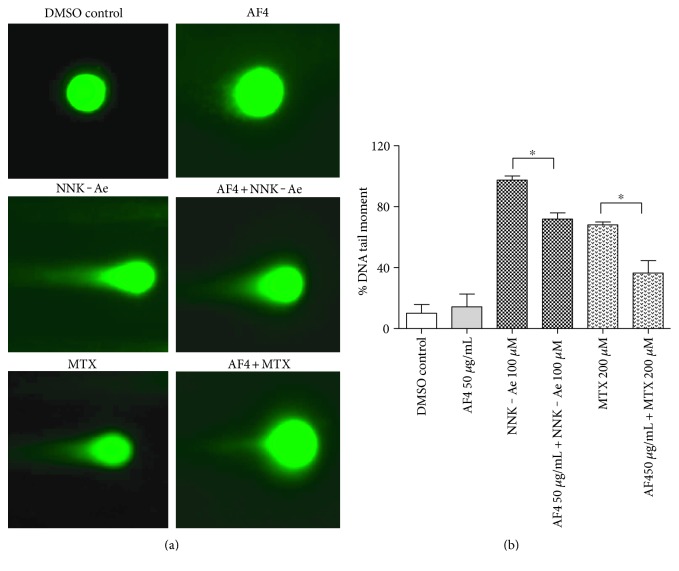
(a) DNA tail damage in BEAS-2B cells exposed to either carcinogen alone or in combination with pretreatment of AF4, as assessed by comet assay. (b) Quantification of DNA tail damage using OpenComet freeware. At least 50 comets for each sample were analyzed and examined with a fluorescence microscopy under 40x. Experimental values presented as mean ± SD of *n* = 3 independent experiments. ∗ indicated statistical difference at *P* ≤ 0.05.

Comet assay was used to measure the DNA strand breaks in an individual eukaryotic cell and got multiple applications such as monitoring environmental contamination with genotoxins, human biomonitoring and molecular epidemiology, DNA damage, and repair studies [25]. After the treatments, DNA tail damage was evaluated as the migration of DNA from the nucleus and the data was quantified and depicted in Figures 5(a) and 5(b). Untreated cells (DMSO control) and AF4-treated cells retained their cellular integrity, and their percentage tail damage were <15%. Similar results were also observed for untreated PC12 neuronal cells [26]. BEAS-2B cells treated with either NNK-Ae or MTX showed a higher percentage of DNA damaged tails (97.4% and 68.0%, respectively), and AF4 pretreatment significantly (*p* ≤ 0.05) reduced the length of percentage tail damage, as quantified from at least 50 comet cells. NNK-Ae-treated cells showed the highest DNA tail damage compared to MTX treatment at identical concentration and time.

### 3.6. AF4 Inhibits DDR Signaling and Facilitate Repair Mechanisms

**Figure 6:**
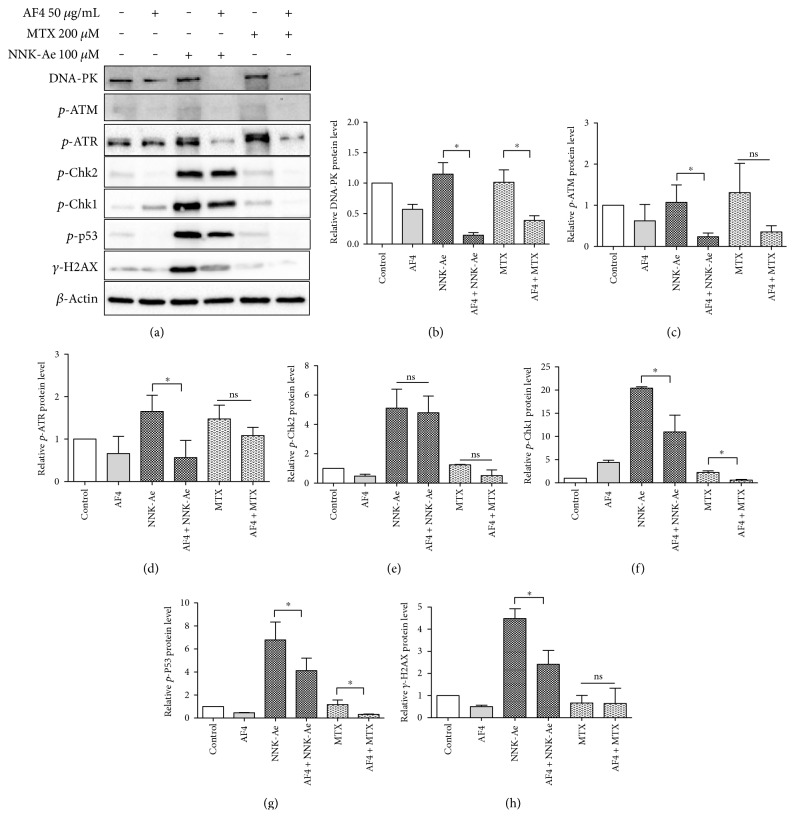
(a) Effects of AF4 on various DDR signaling proteins exposed to NNK-Ae or MTX as assessed by western blotting. (b), (c), (d), (e), (f), (g), and (h) The relative amount of each protein expression levels (DNA-PK, p-ATR, p-ATM, p-Chk2, p-Chk1, p-P53, and *γ*-H2AX) with respect to beta-actin loading control, quantified from at least 3 independent experiments. ∗ indicated statistical difference at *P* ≤ 0.05 with mean ± SD. ns: nonsignificant.

We further investigated the mechanism of action of AF4 to render protection against NNK-Ae- and MTX-induced toxicity in BEAS-2B cells by analyzing signaling proteins involved in DDR process. The phosphorylation levels of ATM, ATR, and DNA-PK were studied using western blotting (Figure 6()). Cell cycle check point kinases Chk1 and Chk2 and tumor suppressor protein p53 were also analyzed and quantified (Figure 6()). ATM/ATR mutations are the primary causes for DNA damage, and they act upstream of p53 sensors and sense DDR functions to the cells [9]. Treatment with NNK-Ae and MTX augmented DDR signaling and ATR phosphorylation (serine 428) in BEAS-2B cells with respect to control cells. However, we did not observe any phosphorylation of ATM protein at identical dosages and time. Expression of DNA-PK level was found to be the same with untreated control or carcinogen-treated cells. Interestingly, pretreatment with AF4 downregulated DNA-PK protein with respect to control cells. Effector proteins like Chk1, Chk2, and p53 were found to be phosphorylated in NNK-Ae-treated cells. In contrast, MTX treatment did not induce these signaling proteins. Pretreatment with AF4 showed significant (*p* ≤ 0.05) reduction in the phosphorylation of ATR, Chk1, and p-53 levels in NNK-Ae-treated cells. We also observed a significant inhibition of *γ*-H2AX protein in AF4-pretreated cells prior to NNK-Ae treatment. Overall, our data showed that pretreatment with AF4 significantly attenuates DDR proteins especially challenged against NNK-Ae genotoxicity.

**Figure 7:**
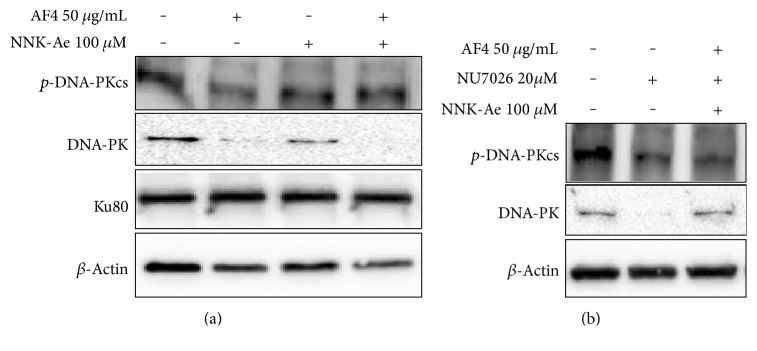
(a) Effects of AF4 on DNA repairing proteins (DNA-PK, p-DNA-PKcs, and KU80) challenged with NNK-Ae alone or in combination. (b) Inhibitory effect of NU7620 (20 *μ*M for 30 mins) against DNA-PK expression on BEAS-2B cells treated alone or with a combination of AF4 and NNK-Ae.

Further, to investigate whether AF4 facilitates DNA repair mechanisms in vitro, we also tested for proteins such as p-DNA-PKcs and KU80 with AF4-pretreated cells prior to NNK-Ae treatment (Figure 7(a)). Interestingly, AF4 reduced DNA-PK level either when treated alone or in combination with NNK-Ae but activates p-DNA-PKcs at the T2609 position. The phosphorylation level of DNA-PKcs was found to be comparatively higher in AF4-pretreated cells than in NNK-Ae-treated cells, indicating its DNA repairing potential. Further to confirm this, we have used NU7026 that inhibits DNA-PK protein expression [27]. Treatment with NU7026 on BEAS-2B cells for 20 *μ*M (30 min) almost eliminated DNA-PK protein in these cells (Figure 7(b)). An autophosphorylated DNA-PKcs protein was observed in untreated cells, and treatment with inhibitor reduces the DNA-PKcs level even in AF4-pretreated cells. This preliminary result further confirms that AF4 pretreatment will facilitate the cells to phosphorylate DNA-PKcs which is essential in NHEJ repair mechanism.

## 4. Discussion

Aberrant mutations in the genome of an organism caused by increased exposure to a carcinogen often lead to a condition called genomic instability. Even low-dose chemicals or environmental exposure can induce DNA damage especially when there is a failure in proper DNA repair mechanism [28]. Due to the excessive ROS, a disturbance in natural antioxidant defense system is expected with damage to all biomolecules, including nucleic acids. Antioxidant-rich diet and nutraceutical supplements can be a good therapeutic strategy to overcome oxidative nucleic acid damage [29]. Hence, in this study, we have evaluated the apple flavonoids, which are a rich source of antioxidants [17, 30, 31] against various carcinogen-induced DNA damage in BEAS-2B cells. We also aimed to study the underlying mechanism of AF4's effects in DDR and repair process followed by DNA damage.

Our previous studies have demonstrated the selective cytotoxicity of AF4 to induce cell death in cancer cells without altering physiological functions of normal cells, including primary human hepatocytes (NHEPS), primary rat hepatocytes (RTCP-10), and primary lung cells (WI-38) [17]. To expand this knowledge, we have analyzed the impact of different doses of AF4 on the viability of BEAS-2B cells and observed that 50 *μ*g/mL maintains cellular integrity with more than 80% viability even after 24 h treatment. However, higher doses were found to reduce the viability considerably and also reported by others [32], suggesting the hormetic effects of dietary flavonoids [14, 33]. Hence, we have chosen 50 *μ*g/mL for evaluating the protective effects of AF4 in BEAS-2B cells. NNK, NNK-Ae, MTX, and cisplatin were used to induce DNA damage in BEAS-2B cells since we and others have observed that these carcinogens can significantly reduce cell viabilities of normal cells by enhancing ROS levels and cell death mechanisms [34, 35]. Pretreatment of AF4 significantly reduced toxic effects of these carcinogens but not for cisplatin treatment. This could be because of an increased intracellular antioxidant enzyme (SOD and small molecules or proteins, as observed in AF4-pretreated groups, which could possibly play a role in scavenging these ROS and helped the cells to mitigate the oxidative stress. Flavonoids are well-known for their ROS scavenging potentials [14, 36], and similar effects for apples were reported by many investigators [15, 16, 31].

Histone posttranslational modifications are one of the earliest events in DSBs and are often characterized by remodeling of chromatin organization [37]. The carcinogens used in this study were observed to modulate posttranslational mechanisms of histone proteins even at 3 h of exposure. This observed toxicity could possibly due to the interstrand crosslink-induced DSBs that are produced at replication forks and are largely responsible for observed *γ*-H2AX foci in carcinogen-treated cells [38]. Each focus assumed to represent a single DSB [39]. However, pretreatment with AF4 inhibited the reorganization of histone variants that regulates DNA methylation. This could account for the similarities in protective effects of both at histone and DNA fragmentation, which appeared to be sensitive tools for analyzing DNA lesions. DNA fragmentation is considered as the hallmark of cell death mechanisms and an irreversible event that commits the normal cell to die [20]. AF4 was found to protect this phenomenon in BEAS-2B cells against NNK-Ae and MTX toxicity. Flavonoids are known to exhibit these protective potentials against various genotoxicity as evident from various studies [14, 29, 40, 41]. The fragmentation level observed in untreated cells could be because of the normal mechanism of the body to dispose large fragments of DNA from dying cells, which may be critical in maintaining normal tissue homeostasis [42].

Consequently, quantitative analysis was carried out by using comet assay to understand the extent of DNA damage caused by carcinogenic factors. Increased comet tails indicated the induction of DSBs through excision followed by resynthesis and ligation of fragments. A significant inhibition of DNA tail damage was recorded in AF4-pretreated cells when compared to carcinogen treatments, further, substantiate the potential of AF4 to render DNA protection in BEAS-2B cells. Taken together, we speculate that these protective effects are mainly due to either AF4's antioxidant properties or its ability to stimulate DNA repair enzymes. Polyphenols such as luteolin, quercetin, and rosmarinic acid have shown similar effects to protect DNA damage against oxidative stress in neuronal cells [14, 26]. A recent study has also shown that sesaminol, a lignin from sesame seeds with increasing activities of catalase and SOD, protects BEAS-2B cells against DNA damage caused from cigarette smoke extract [43]. All these studies confirm that plant polyphenols with antioxidant activity could counteract the toxic effects of carcinogens and may help to maintain genomic stability.

In order to test our hypothesis, we further investigated the molecular mechanism of DSBs induced by NNK-Ae or MTX, since it is crucial to identify therapeutic targets during drug discovery process. The recruitment of DDR factors to DSBs was analyzed by immunoprobing against different proteins (ATM, ATR, DNA-PK, Chk1, Chk2, and p53). ATM/ATR mutation plays a key role in surveillance of genomic integrity along with signal transducers [38]. ATM-Chk2 or ATR-Chk1 are the two common pathways that get activated during DSBs and ultimately triggers p53 [44]. Our data showed that NNK-Ae induces DSBs through the phosphorylation of ATR and not ATM in BEAS-2B cells. ATR is the major kinase activated during a replication stress and plays a key role in “S” phase cell cycle arrest [11]. Effector proteins such as Chk1, Chk2, and p53 also became activated by NNK-Ae treatment. However, MTX did not induce these proteins in BEAS-2B cells. We speculate that lower dosage and exposure time for MTX may be ideal for inducing early events in DSBs but may not be sufficient to activate a cascade of effector proteins. Moreover, MTX is also known to have therapeutic applications when used at lower doses [45]. We have also observed the phosphorylation of DNA-PK at T2609 loci which is the most common target for its activation [46]. ATM/ATR often thought to coregulate DNA-PK expression in DSBs, but their choice of involvement still remains inconclusive [4, 11, 46].

Consistent with our immunofluorescence data, exposure to NNK-Ae triggers the phosphorylation of *γ*-H2AX as observed in western blot, further confirms the reorganization of histone proteins during DSBs. One hour of AF4 pretreatment significantly inhibits ATR/Chk1/p53/*γ*-H2AX signaling, suggesting the mechanism of protective effect possibly through ATR-dependent manner. Further, we also evaluated AF4's involvement in DNA repair mechanisms. AF4 slightly activates DNA-PKcs along with coexpression of KU80 protein in NNK-Ae-treated BEAS-2B cells. The activation of DNA-PKcs primarily enhances NHEJ repair mechanisms [4]. This effect of AF4 was confirmed by using a DNA-PK inhibitor, NU7026. However, more studies are required to claim DNA repairing efficacies of AF4 against NNK-Ae exposure. Overall, our study enlightens to be the first step in evaluating apple flavonoids against oxidative damage induced by carcinogens in bronchial epithelial cells.

In summary, our studies showed that preexposure of apple flavonoids protect BEAS-2B cells challenged against various carcinogens, especially nicotine-derived nitrosamine ketones, by inhibiting DDR signaling and initiate DNA repair mechanisms. Further studies can also give insights to understand the active constituents of AF4 that can also be developed as potential therapeutic adjuvants to reduce the side effects of various cytotoxic or genotoxic chemotherapeutics.

## Abbreviations

AF4: Apple flavonoid fraction
ATM: Ataxia telangiectasia mutated
ATR: ATM-Rad3-related
BEAS-2B: Normal human bronchial epithelial cells
BEGM: Bronchial epithelial cell growth medium
CHK: Check point kinases
DDR: DNA damage response
DMSO: Dimethyl sulfoxide
DNA-PK: DNA-protein kinases
DSBs: DNA double-strand breaks
HR: Homologous recombination
IF: Immunofluorescence
MDC1: Mediators of DNA damage check point 1
MTX: Methotrexate
NHEJ: Nonhomologous end joining
NNK: 4-(Methylnitrosamino)-1-(3-pyridyl-d4)-1-butanone
NNK-Ae: NNK acetate
PI3K: Phosphatidylinositol-3-kinase
ROS: Reactive oxygen species.
